# Effect of Moringa oleifera‐supplemented diets on cognition, neurochemicals, and inflammation makers in the Brain diabetic‐encephalopathic rats treated with Acarbose

**DOI:** 10.1002/alz.091219

**Published:** 2025-01-09

**Authors:** Idowu Sunday Oyeleye, Ganiyu Oboh, Omamuyovwi Ijomone

**Affiliations:** ^1^ Federal University of Technology, Akure, Akure Nigeria; ^2^ Federal University of Technology, Akure, Ondo Nigeria; ^3^ The Federal University of Technology Akure, Akure Nigeria

## Abstract

**Background:**

The progression of diabetes mellitus (DM) has been associated with changes in brain structure and function, often referred to as “diabetic encephalopathy,” which is characterized by cognitive and neurochemical dysfunction, and identifiable structural changes in brain imaging. This study investigated the effect of Moringa leaf‐supplemented diets (MLSD) on cognition, acetylcholinesterase (AChE), adenosine deaminase (ADA) and arginase activities, reactive oxygen species (ROS), total‐thiol (T‐SH), inflammatory cytokines (TNF‐α, NF‐κB, IL‐6, and IL‐10) levels, caspase‐3 expression, and nuclear factor erythroid 2–related factor‐2 (Nrf2) levels in the brain of DM rats treated with 25 mg/kg bwt acarbose (ACA).

**Method:**

The normal control (NC) rats and diabetic rats were grouped as follows: NC rats, untreated DM rats, DM rats plus ACA, DM rats plus ACA and 2% MLSD, and DM rats plus ACA and 4% MLSD. The NC, DM, and DM plus ACA rats were fed basal diets (i.e., without test samples), while other groups received the corresponding test samples for 28 days. On the last day, cognitive function was assessed using the Morris water Maze and Y‐maze tests. AChE, ADA, ROS, T‐SH levels, TNF‐α, NF‐κB, IL‐6, IL‐10, caspase‐3 expression, and nuclear factor erythroid 2–related factor 2 (Nrf2) levels were measured in the brain.

**Result:**

The untreated DM rats had altered cognition, increased ROS, TNF‐, NF‐B, and IL‐6, caspase‐3 expression and NrF2 levels, and AChE, ADA, and arginase activities, but decreased T‐SH and IL‐10 levels when compared to the NC group. These, however, were reversed in DM rats fed MLSD but were highly significant in DM rats co‐treated with MLSD plus ACA.

**Conclusion:**

The findings suggested that a combination of MLSD plus ACA could be explored to combat DM‐induced cognitive dysfunction

